# L-VISP: LSTM Visualization for Interpretable Symptom Prediction in Patient Cohorts

**DOI:** 10.1111/cgf.70314

**Published:** 2026-03-19

**Authors:** C. Floricel, Y. Wang, A. Wentzel, C. D. Fuller, G. E. Marai, M. E. Papka, G. Canahuate

**Affiliations:** 1 University of Illinois Chicago, Chicago, IL, USA; 2 University of Iowa, Iowa City, IA, USA; 3 University of Texas, Austin, TX, USA; 4 Argonne National Laboratory, Lemont, IL, USA

**Keywords:** medical XAI, LSTM modeling, multidisciplinary visual analytics, temporal visualization

## Abstract

Symptom modelling in head and neck cancer is challenged by the complexity of heterogeneous patient data, leading to an interest in deep learning approaches. Although Long Short-Term Memory Networks (LSTMs) have shown great results in patient risk prediction, their low interpretability requires data modellers to collaborate with clinical experts to validate the results. We present L-VISP, a human–machine solution that uses visual analytics for LSTM modelling in clinical research. L-VISP uses custom visual encodings to make multiple LSTM variants interpretable, supporting a full range of analysis, from understanding model operations and evaluating performance to interpreting results in a clinical context. We evaluate L-VISP with data modellers and a clinical oncologist and present the takeaways from this multidisciplinary collaboration.

## 1. Introduction

Personalized head and neck cancer (HNC) care focuses on creating treatments tailored to individual patients based on cohort characteristics from similar patients. Unfortunately, cancer treatment often results in numerous side effects, which differ between patient cohorts and can last for a long time post-treatment. As a result, clinicians are collaborating with data modellers to understand treatment-related symptoms that appear or persist post-treatment, to predict adverse outcomes and to stratify patients into high- and low-risk cohorts. One of the significant challenges in post-treatment research is posed by the scarcity of cohort data, imposed by the patient monitoring protocol [CMW*00]. Patients are closely monitored during treatment, when they come to the clinic to receive the prescribed doses, as opposed to post-treatment, when they come for biannual follow-up checkups [HGE*17]. As a consequence, post-treatment patient data are collected less often, posing a challenge in outcome prediction. Long Short-Term Memory Network (LSTM) methods have demonstrated excellent results for temporal patient outcome prediction, surpassing traditional statistical and machine learning methods, and have also gained attention in HNC symptom risk prediction [WCVD*21, WVDM*23]

Post-treatment symptom risk prediction is a multidisciplinary field where data modellers collaborate with clinicians to model patient outcome risk, but this modelling often suffers from low interpretability. This is especially true when supervised black-box models, such as LSTMs, are used. Visual analytics can support this research; however, it needs to consider the differences in the mental models of the users. For example, clinicians are more interested in the actionable interpretation of the modelling outcomes and in the accuracy of the methods, which can be applied when treating new patients. Data modellers, on the other hand, are also interested in understanding the mechanisms behind the model’s decisions and tools that help them refine and debug modelling approaches. Moreover, post-treatment symptoms can result from the cumulative effects of various factors [OHS*16, VdBvdSvdL*21], such as treatment-related complications or patient-specific health and lifestyle changes following treatment. Symptoms can also be associated with each other, either due to direct influence, or due to shared root causes. Consequently, there is a growing need for analytical tools that support collaborative cohort modelling through workflows that enable experts to interpret machine-derived (modelled) results with real patient data.

Although data visualization is a valuable tool for supporting analytical tasks, it must overcome several challenges in the context of post-treatment symptom prediction. To effectively interpret LSTM model behaviour, data visualization must integrate diverse data facets from heterogeneous cohorts and support data modellers’ and clinicians’ analytical tasks. Specifically, data visualization needs to compare multiple cohorts of interest to understand the impact of the modelled risk, support cohort stratifications by levels of risk to better understand prediction results and blend cohort characteristics with results from different models to gain a deeper understanding of risk categories. Notably, LSTM symptom prediction visualization needs to overcome the cognitive burden associated with the high information density of LSTM models.

To address these challenges, we introduce L-VISP, a visual analytics system developed for and with data modellers, with clinicians as secondary users, which supports post-treatment risk modelling in HNC patients. Below are this work’s main contributions:

C1. The domain characterization, developed alongside domain experts, of an application that targets model explainability in LSTM predictive analysis for head and neck cancer cohorts in collaborative clinician—data modeller settings, with a description of the modelling problem and design requirements;
C2. Data modelling with unsupervised and supervised approaches to stratify patient cohorts by risk using temporal clustering and to model symptom severity using LSTM methods;
C3. A human–machine system that is a collaborative bridge for data modellers and clinicians in clinical research. The system blends data visualization with data modelling to interpret model outputs on multivariate, temporal patient cohorts. The system uses custom visualizations to blend different facets of the ground-truth data and outcomes from multiple models and to validate the models on machine-generated (clusters) or user-defined patient cohorts.
C4. The evaluation of the resulting system by modellers and a clinician, along with a thematic analysis of their feedback, and lessons learned from this multidisciplinary collaboration.

## 2. Project Setting

In this section, we describe the project setting, task analysis and the data that we modelled and visualized.

This work is part of an interdisciplinary collaboration between three research groups located at different sites: two data modellers with experience in symptom modelling, four visual computing researchers with modelling experience and a radiation oncology expert with clinical and modelling experience. All collaborators are co-authors of this work. The application domain focuses on scientific and analytical research support for exploratory data analysis, model evaluation, hypothesis generation and research communication through insightful visualizations. As a result, we used an Activity-Centred-Design (ACD) [Mar18] paradigm to characterize the domain application, which is particularly suitable for scientific research, especially due to the scarcity of trained domain experts and the importance of slow thinking [Kah11] for scientific research. ACD prioritizes the user activity over the number of users.

Through a series of iterations, the visual computing team and the data modellers held weekly meetings to define functional specifications and to prototype the interface. This was an iterative process that evaluated incremental designs, starting with paper prototypes, then narrowing down proposed designs alongside data modellers, and finally moving to digital prototypes. Before evaluating the final system, the team met with the clinician to demonstrate the system. The clinician was not part of the design process, but provided feedback during the domain characterization and was part of the evaluation of the final system. The data modellers were part of the domain characterization, design and evaluation stages.

### 2.1. Task analysis

L-VISP was built to primarily serve data modelLers in cancer symptom research. The system evaluates two LSTM variants for symptom risk in the context of patient cohort data. Our modelLer collaborators have experience in ML approaches for predicting patient outcomes, but they treat the predictive model as a black box. Thus, they needed an overview of the model’s behaviour and its sensitivity to input variations, as well as an explanation of the output. Through the ACD paradigm, the regular meetings revealed the modellers’ process, which involved running multiple scripts with numerous parameters and verifying each output plot individually. However, this process made it difficult to assess multiple outcomes concurrently on a desired cohort. Furthermore, the group was interested in having the clinician collaborator validate the modelling results. As a result, part of L-VISP’s front-end targets clinicians with modelling experience. These front-end components support the clinician’s interpretation of symptom predictions on cohorts of interest and are dedicated to one of the main user activities, which include both clinician and modeller tasks, presented in detail in Section 4.

We identified several key tasks and, following the ACD framework, grouped them into two main activities: the first one for analysing model performance and behaviour for the patient population which is stratified by symptom severity using temporal clustering, and the second one for analysing the model performance for a target, user-specified patient cohort. Other works in LSTM modelling visualization focus on either finding patterns in prediction trajectories [HSYZ24], comparing predicted data against ground-truth data to find prediction errors [CWO*24] or visualizing model hidden states [SGPR17] to understand the model’s decisions. Our activities, however, need to support a combination of these tasks. In addition, we compare modelling results for two patient cohorts and predictions between data items (*i.e.* symptoms), and visually combine the results from two LSTM variants. We present below the two activities (A), which are composed of several visualization tasks (T):

A1 Stratified model evaluation for patient clusters.
  T1. Validate model predictions by comparing ground-truth symptoms to predicted symptom ratings, to test the model accuracy. For example, to check how accurately the model predicts that taste issues remain severe at the end of the patients’ monitoring program, the data modeller needs to compare ground-truth ratings with the predicted ratings.
  T2. Analyse the model behaviour by exposing the model memory and evaluating results on different cohorts, to understand underlying mechanisms. For example, to understand how the model learned to make the predictions for the end of the monitoring protocol for taste, which could be by either learning from past taste ratings or relying on the past ratings from other symptoms, the data modeller needs to analyse the weighted associations between taste and all symptoms. To further analyse whether the model shows behaviour changes for patients with various symptom burden, the modeller needs to evaluate these results on different patient clusters.
  T3. Compare model results between patient clusters by analysing prediction trajectories and performance metrics, to find model deficiencies. For example, to understand whether the predictive model fails to predict the symptoms for the patient cluster with mild overall symptom burden, but succeeds in predicting symptoms for the cluster with moderate symptom burden, the data modeller needs to compare the prediction performance metrics (*e.g.* AUC, F1 scores) between these clusters. To add more context to these metric results, they need to further analyse the similarity between the predicted trajectories and ground-truth symptom trajectories for the patient clusters.
A2 Targeted model evaluation for user-defined patient cohorts.
  T4. Examine input–output relationships in the model by evaluating predictions under different cohort attributes, to test the model’s robustness. For example, to examine how stable the model results are under different conditions, the data modeller needs to examine the predictions (outputs) for patient cohorts with different attributes, such as female patients, or patients with swallowing difficulties (input condition is high swallow severity) and so on.
  T5. Evaluate model performance for a given cohort by comparing predictions between desired cohorts against the rest of the patient population, to test the model accuracy and find cohorts for which the model predicts negative outcomes. For example, to find at-risk patients predicted by the model, the clinician needs to evaluate if the predictions for cohorts that they know are susceptible to negative outcomes, such as patients with dysphagia/swallowing difficulties, show significantly different predictions from the rest of the patients.
  T6. Find model connections to symptom severity by evaluating predictions under different symptom severity thresholds, to test the model’s accuracy and find symptoms linked to high risk of negative outcomes. For example, to analyse if the model can flag patients who will cross the severe threshold for critical symptoms, the clinician needs to evaluate the predictions for patients with medium-to-severe symptoms, such as patients with medium taste problems, which are some of the most prevalent in HNC.

Although these activities were extracted in accordance with data modellers’ needs, A2 was documented to support clinician analysis as well. The clinician provided occasional feedback on the above-mentioned tasks during the domain characterization phase, which helped to define the activities. Our clinician collaborator was not interested in understanding the mechanisms of the LSTM (A1), but wanted to analyse outcomes on cohorts of interest (A2). Consequently, L-VISP becomes a collaborative tool that facilitates joint workflows. First, the data modeller uses A1 to debug and gain confidence in the LSTM model. Afterwards, the modeller and clinician can collaborate in A2 to validate the model’s predictions against clinical intuition and explore outcomes for desired cohorts.

### 2.2. Data

The data were collected from a cohort of 937 head and neck cancer patients from the MD Anderson Cancer Center in Texas, treated with radiation therapy (RT). We refer to the whole dataset as the patient population, and to a subset of the patient population as a patient cohort. The data include clinical and treatment attributes, and patient-reported symptom ratings. Clinical data include demographics such as age (quantitative), gender and smoking status (nominal); diagnostic attributes such as tumour size and lymph node stage (ordinal), tumour site (nominal) and additional treatments: induction therapy (IC), concurrent therapy (CC) and neck dissection surgery (ND) (nominal). The clinical data are visualized to support the analysis of the patient clustering and symptom prediction. We use symptom ratings and treatment attributes for symptom prediction, and symptom ratings for patient clustering.

#### 2.2.1. Ground-truth symptom burden

The MD Anderson Cancer Center studies symptom severity in patients through a quality-of-life monitoring program. The program involves patient-reported outcome (PRO) questionnaires based on the MD Anderson Symptom Inventory-Head and Neck Module [CMW*00], also known as MDASI-HN. This 28-symptom questionnaire is used for clinical and research purposes, in which patients are asked to rate symptoms using a 0–10 scale, from *‘not present’* (0) to *‘as bad as you can imagine’* (10) (Table 1). Symptoms are split into three categories: HNC-specific, general cancer and daily interference symptoms. The PRO data are temporal and multivariate, collected before, during and after treatment throughout a total of 12 time points. Over half of this data collection happens during treatment, when a spike in symptom severity is expected due to treatment toxicity. Our data analysis evaluates modelling results based on several rating thresholds with clinical significance: ≥ 1 <3 (labelled as ≥1 in the front-end) for mild symptoms, ≥ 3 <5 for mild-to-moderate symptoms (labelled as ≥3 in the front-end) and ≥5, which clinically stand for moderate-to-severe symptoms. These thresholds were chosen based on our clinician collaborator feedback, previous clinical research on the MDASI-HN questionnaire [ASN*20, RMC*08, RMC*07] and our previous projects with the questionnaire [WCVD*21, BFVD*21, FNB*21, FWM*23, WFC*23].

In this work, we aim to identify patients who experience late symptoms well after treatment has concluded. As a consequence, we use the post-treatment time points from the PRO symptom data to predict late symptoms, and the baseline time point to have a baseline for the post-treatment predictions. We omit the intermediate, during treatment, time points to not influence the predictions. Namely, we use the symptom data before treatment, or baseline (B), at the end of treatment (W0), and during post-treatment at: 6 weeks (W6), 6 months (M6) and 12 months (M12) (Table 1). Although we used all 28 symptoms to cluster patients by symptom severity, we performed LSTM modelling and used visual analysis to evaluate model’s decisions for the nine HNC symptoms: swallow, speech, mucus, taste, constipation, teeth, mouth sores (mucositis), choking and skin problems. We used all 28 symptoms in patient clustering to capture a comprehensive view of symptom burden and patients’ variability. The selection of the nine HNC symptoms for LSTM modelling followed, driven by clinical relevance and the need for focused analysis on symptom categories. Our collaborators showed particular interest in the HNC subset before extending the analysis to the daily interference and general cancer categories. The modellers aimed to present results to the clinician for this symptom subset to inform planning for further patient cohort modelling projects. Visualization considerations for supporting the analysis of nine symptoms include the limited real estate of the front-end and the need to limit the cognitive load due to high data density, aspects discussed in Section 6.

#### 2.2.2. Predicted symptom burden

In addition to the ground-truth patient data, we visualize the results of our models (Figure 3). The data modellers used the bidirectional LSTM (Bi-LSTM) [WCVD*21] for symptom severity prediction and the Interpretable Multi-Variable LSTM (IMV-LSTM) [GLAF19] for Bi-LSTM modelling understanding. As a result, we used visual analytics to support the evaluation of the Bi-LSTM predictions, the Bi-LSTM performance metrics and the IMV-LSTM features; all described in detail in Section 4.1. Additional cohort modelling is done with the Time2Feat time-series clustering method [BDBGT22], which the modellers used to extract three patient clusters with different symptom severity thresholds. These results are integrated into our visualization system as well.

## 3. Related Work

In this section, we describe the related work relevant to this research, namely, visualization methods for multivariate, temporal medical cohorts, visualization for medical applications that tackle interpretable ML and visualization for LSTM model interpretation.

### 3.1. Multivariate, temporal cohort medical visualization

Visual analytics in medical applications often supports patient analysis by integrating attributes from health records [GXZ*17, HWZ18, Rai19, BSM*15, RAS20, CWCN11], which requires human interpretation of heterogeneous data. Visual analysis for patient cohorts tackles statistical analysis for cohort history comparison [GS14, ZGP15, PMR*96] and outcome analysis [WG11, KCK*18, RSN*19]. Visual encodings for these applications span from histograms [BPW*21, JLC*24], matrices [MD*15, DPSS16], radial-charts [GDM*19], to time-series plots [GS14, JCG*20, KLJ*25]. In personalized medicine, the focus is on analysing the current patient in relation to its cohort [YCB*22] and identifying outlier patients, understanding their differences and similarities to the rest of the population. This analysis is often visually supported by scatterplot projections [FWM*23, WHL*20, HSDH*24]. However, scatterplot projections can suffer from visual clutter and glyph overlap in large populations, failing to support the precise, nonoverlapping selection of individual patients required for our detailed cohort analysis. We propose a matrix-based encoding for the same tasks, to project in 2D the patient population and to highlight desired cohorts.

Due to its temporality, patient cohort data have been used in different modelling approaches, such as mining temporal patterns [WMH*21, CCDW17, FNB*21], clustering patient trajectories for cohort summaries [GXZ*17, MKK*19] and predicting prognosis and outcomes [GGJ*21, CCDW17, WGGP*11]. Summarization of cohort time-series has been supported by various representations, ranging from matrix-based [DPSS16] to flow-based [GS14, WG11] and PCP-based [MNB*21, BPW*21] approaches, as well as customized timeline visualizations [PMR*96, BPW*21, GXZ*17]. Although previous methods can summarize general trends, they do not support simultaneous clustering and comparison of trajectories between ground-truth and predicted values across different cohorts, which is essential for validating the model’s temporal accuracy. We propose clustered lineplots to support trajectory comparisons between cohorts, accompanied by dendrograms to uncover time-series similarities. We utilize grid-based encodings to compare the temporal distribution of modelled and ground-truth values.

### 3.2. Visualization for interpretable ML in medical applications

Visualization for explainable AI (XAI) or machine learning (ML) medical applications seeks to enhance the human interpretation of machine-generated outputs in patient cohorts, *i.e.* the clinician’s interpretation of model-computed outputs [AB18, JCG*20, LWQ24, MZWOJ19, HSB*20, WCM*25, BSL*25, CZJ*24]. Related work has proposed visual analytics approaches that incorporate ML outputs in application areas, such as patient prognosis [WG11, LLC*24, GXZ*17], survival prediction [LLM23, WAZ*25] and treatment outcome prediction [GGJ*21, GS14, KCK*18, KPK16]. In this project, we tackle treatment outcome prediction by visualizing symptom predictions. Some proposed work compares modelling results between cohorts [WFC*23, MD*15], or analyses the results of a sub-cohort/cluster against the rest of the population [YCB*22, JLC*24]. Although these approaches support either cohort comparison or cohort analysis in isolation, they fail to provide a system that supports both the validation of machine-generated clusters and the exploration of user-defined cohorts against the rest of the population. In L-VISP, we support both types of analyses through two main user activities: visual analysis of modelling results on a user-defined cohort against the rest of the patients and visual comparison of modelling results between two patient clusters.

Visual analytics for black-box models is a challenging domain due to the models’ inherently opaque nature. Some tools have been proposed to leverage the collaboration between a clinician and an AI model [KCK*18, WAZ*25, KLJ*25]. However, many times, clinicians collaborate with data modellers to interpret cohort modelling [FMCM*21, HSB*20, LLM23]. Our previous work has focused on tools that support multidisciplinary clinician–modeller collaborations for patient outcome and risk analysis [FNB*21, WFC*23, FWM*23, WAZ*25]. However, the functionality of that work failed to satisfy considerations that were imperative to this project. Previous work did not support long-lasting symptom prediction analysis, as the predictions relied on during-treatment symptoms and on finding associations between the during-treatment and post-treatment stages [FWM*23, FNB*21]. In this work, we switch to black-box modelling using LSTM methods to predict long-lasting symptoms post-treatment. Moreover, previous work has either focused on solutions dedicated to both clinicians and modellers [FWM*23, WFC*23], or on solutions dedicated to the clinician’s workflows [FNB*21, WAZ*25]. Due to transitioning to more opaque modelling (*i.e.* using black-box models), we switch our focus to the data modeller tasks, where the clinician is a secondary user. This implies design choices that can help the modeller to unravel the underlying mechanisms of the model, as well as help to validate the predictions with a clinician. Another consequence of switching to black-box modelling was the need for a more guided analytical workflow, which would separate the needs of the data modellers from the needs of the clinicians. Our previous work failed to guide users in creating desired workflows, by either proposing front-ends with too many configurations [FWM*23], or no configurations at all [FNB*21]. In this work, we visually separate user workflows through multiple front-end panels. Another consideration for this project was the need to verify modelling results on cohorts of interest by both the data modeller and the clinician. Previous work did not provide this flexibility, supporting either the visual analysis of pre-defined patient clusters [FWM*23] or cohorts defined by a small set of clinical attributes [FNB*21]. In this work, we consider the analysis of both patient clusters, which is dedicated to the data modeller tasks, and of user-defined cohorts based on a larger set of attributes, which is of main interest for the clinician tasks.

### 3.3. LSTM visualization

LSTM-based models are a deep learning method that can deal with complex time series while showing excellent results for a variety of applications, spanning from finance and economics to epidemiology and other biomedical applications [LRBB*23, HSYZ24, MXC*19, ZDXR20]. Past work in XAI visual analytics for explaining LSTM prediction models has supported the understanding of model performance by exposing models’ hidden state dynamics, evaluating performance statistics and comparing modelling outputs with ground-truth data [HSYZ24, CWO*24, CXZ*24, WXG*24, SGPR17]. These works have experimented with matrix-based visualizations, as well as with timeline and flow-based visual representations of the predicted results [SGPR17, HSYZ24]. Existing LSTM visualization tools generally focus on one analytical task and fail to support the comprehensive workflows required to inspect the model’s underlying mechanisms while validating the model’s outcomes. We use similar visualization techniques and combine multiple essential analytical tasks. Specifically, we expose the model’s hidden state dynamics, evaluate the model’s performance and compare predictions against ground-truth data. Another difference from previous work is that we aim to visually combine the outputs of two distinct and complementary LSTM models (*i.e.* the Bi-LSTM and IMV-LSTM). Finally, unlike some of the previously mentioned work, our study aims to evaluate and compare model results across patient cohorts. Namely, it supports model evaluation on cohorts that are either user-defined (*e.g.* female patients under 50) or extracted after cluster modelling (*e.g.* patient cluster with severe symptom ratings).

Our collaborators have previously experimented with unsupervised rule mining [BFVD*21, FNB*21], clustering [FWM*23] and LSTM-based modelling [WCVD*21, WVDM*23] to predict symptom risk and to find associations in multivariate patient cohorts. In this work, we do this with supervised LSTM methods, which can help to predict symptom risk in new patients at the beginning of their treatment, without necessitating their temporal records during treatment. As opposed to the previous LSTM modelling approaches for symptom prediction, the methods used in this work utilize Bi-LSTM to capture bidirectional temporal dependencies for improved predictive performance, while incorporating interpretability mechanisms to enhance transparency (with IMV-LSTM).

## 4. System Design

This section presents the back-end, namely the LSTM-based symptom prediction and the patient clustering, and the front-end components that visualize the modelling results. L-VISP uses Python for the back-end and React with D3.js for the front-end. All modelling is computed offline before being loaded into the front-end.

### 4.1. LSTM symptom modelling

Our modelling approach uses a pair of variants from the LSTM family for symptom risk analysis (A1, A2). The first is a Bi-LSTM [SP97], which acts as our primary prediction model. By processing a patient’s timeline in both forward and reverse, it gains a deeper context to forecast future symptoms. Specifically, Bi-LSTM contains two LSTMs that go in opposite directions, which allows them to capture upstream information and additional context at each time point. After running the LSTM in both directions, the hidden states are concatenated, *i.e.* the dimension of the hidden states is doubled, before generating the final output. This ensures that more information is gathered, which improves the final prediction results. The second LSTM model is an Interpretable Multivariate LSTM (IMV-LSTM) [GLAF19], which serves as the explainer, and looks inside the Bi-LSTM to understand how it arrives at its predictions by exposing its memory/hidden states. We applied Bi-LSTM and IMV-LSTM on the nine symptoms of interest.

Our primary goal was to predict long-term symptoms at the 12-month mark (M12), which corresponds to long-term/chronic symptoms. To prepare the model for this task, we first trained the Bi-LSTM to impute missing ratings from earlier and subsequent time points, ensuring it had a complete patient history to learn from before making its final (M12) prediction (Figure 3). Specifically, the modellers first imputed the missing baseline (B) ratings using the mean values of the entire cohort (Figure 3a). In their preliminary work [WVDM*23], they have experimented with other imputation methods [ANI*20], such as a K-nearest neighbours baseline imputation. Compared to the mean baseline imputation, the results have shown a similar AUC performance on the month 12 (M12) prediction. Given the comparable performance, they opted for a mean baseline imputation for the present project.

They then trained the Bi-LSTM model recursively on the preceding time points and let it predict the current time point for all time points (W0-M6) before the last recorded one (M12) (Figure 3a). In their preliminary work [WCVD*21], the modellers have experimented with Multivariate Imputation by Chained Equations (MICEs) [VBGO11], Linear Interpolation and Kalman Interpolation [Kal60] methods to impute missing symptom ratings. However, they proposed an LSTM recursive imputation, which showed more satisfactory results. In this project, they wanted to experiment with the Bi-LSTM variant. Specifically, using three-fold cross-validation, our collaborators used the B-M6 imputed symptoms (Figure 3b) and trained the Bi-LSTM model over two training folds to predict M12 symptom values for all patients in the test fold. Model performance metrics were extracted, including the area under the curve (AUC), F1 score (micro averaging), Precision, Recall and root mean squared error (RMSE) for each symptom.

While the recursive predictions can accumulate errors, we reduce the risk by training each prediction step with an internal validation portion of the ground-truth outcome at the next time point. We use internal validation at every stage before applying the model to the test dataset. In addition, we use cross-validation to ensure that every partition of the data is evaluated, and we use early stopping to maintain stable and generalizable predictions while avoiding overfitting to noise during intermediate steps.

To understand the reasoning behind the Bi-LSTM’s predictions, we used the IMV-LSTM. This second model analysed the complete patient history to determine the temporal importance (*i.e.* which symptoms across the B-M6 time points were most influential) and the variable importance (i.e., which other symptoms or treatments had the greatest impact) for the final M12 prediction. Following the terminology in [GLAF19], we define the feature importance from the IMV-LSTM during the intermediate time points (B-M6) as temporal importance, and the final feature importance predicting M12 as the variable importance. IMV-LSTM defines a hidden state matrix to monitor and obtain both the temporal importance (1) and the variable importance (2) from the LSTM hidden states. By using a mixture-attention mechanism, IMV-LSTM applies temporal attention/importance to the sequence of each variable’s hidden states to obtain a summary of each variable’s history. After that, variable attention/importance is computed from each variable’s history-enriched hidden states. The mathematical definitions of the temporal importance and the variable importance for a given symptom are shown below:

(1) $$A=\frac{1}{M}\overset{M}{\underset{m=1}{\sum }}A_{m};\;A_{m}=\left[\alpha_{1,m},\ldots ,\alpha_{T,m}\right]$$

where *A* is the temporal importance vector computed by taking the average of the attention weights *α* for all the data instances, *M* is the number of patients and *T* is the number of time points preceding the last one (B-M6). In our context, the temporal importance of each symptom is the average of the attention-weight vectors over all the patients. To derive temporal importances for each of the nine HNC symptoms, we trained nine separate interpretable multivariate (IMV)-LSTM models, with each model targeting one specific symptom. For each model, the predictors included three treatmentconditions and all other HNC symptoms except the target symptom. This design allows the temporal importance scores to capture not only the contributions of preceding time points for the same symptom, but also the cross-symptom associations that influence the prediction of the target outcome. In a clinical context, given a symptom, such as pain, the temporal importance reflects how both within-symptom history and other symptom trajectories (*e.g.* swallow, taste, voice, *etc.*) jointly contribute to predicting pain at M12. A high score means the model found a strong influence for a symptom at a time point preceding M12 to predict another symptom’s M12.

(2) $$B=\frac{1}{M}\overset{M}{\underset{m=1}{\sum }}B_{m};\;B_{m}=\left[\beta_{m}^{1},\ldots ,\beta_{m}^{n}\right]$$

where *B* is the variable importance computed by taking the average of the posterior probability *β* for all the data instances (patients) across all input variables, *M* is the number of patients and *n* is the number of input variables (nine symptoms and three treatment conditions). The resulting posterior probability is computed by a soft-max layer of a neural network, whose input combines attention-weighted summary with the hidden state vectors of each variable (symptoms and treatment conditions). In a clinical context, for each predicted symptom (*e.g.* pain), the model calculated the importance score for all other symptoms (*e.g.* taste, voice, choke, *etc.*) and treatments (RT, IRT, ICC). This score represents the influence of the other symptoms and treatments to the final M12 prediction of the target symptom.

The modellers conducted the same analysis for each symptom to obtain the temporal and variable importance. They first removed the symptom to predict from the training data to avoid the target symptom from dominating the variable/temporal importance. After extracting the temporal and variable importance, they obtained relations between each symptom and all the other symptoms. They used the IMV-LSTM’s area under the curve (AUC) score as a quantifier of the strength of the relationships. The higher the AUC, the better the model can distinguish between the positive and negative classes; thus, the more confident we can say that the temporal and variable importance patterns are useful for making accurate predictions.

Both LSTM models were trained using the mean squared error loss function with early stopping. Parameter tuning was performed using an 80/20 data split. The Bi-LSTM model used one recurrent layer with a size of ten and trained with a learning rate of 0.0215 using Stochastic Gradient Descent. The IMV-LSTM used a hidden state size of 128 and was trained with a learning rate of 0.001 and a weight decay of 0.9, using the Adaptive Moment Estimation optimizer. The Bi-LSTM training on an RTX 4080 platform with a three-fold cross-validation required on average 4.2 s per time point and around 17 s in total, while the prediction on the test set was completed in less than 0.1 s. These runtimes indicate that the modelling is computationally efficient and suitable for offline analysis.

### 4.2. Multivariate temporal patient clustering

A key goal for our collaborators was to stratify the patient population based on the overall severity of their symptoms over time (A1). This was needed to find patients with high, medium and low risk of symptom burden. This was a complex task, as each patient’s history involves multiple symptoms that evolve in distinct ways. Our collaborators have experimented with several patient clustering methods before Refs. [MMB*19, FNB*21], which could not capture the multivariate time series nature of our data well. Most temporal clustering methods that consider either univariate or multivariate time series (*e.g.* dynamic time warping [DTW], K-Shape, CSPCA, MC2PCA) [BDBGT22] suffer from poor explainability, and the original data dimensions are lost. As a result, in this work, our collaborators explored Time2Feat [BDBGT22], which is specifically designed for complex time-series and aims to create understandable clusters. This method focuses on interpretable features extracted from time series and uses dimensionality reduction on subsets of features that retain the most information, providing highly interpretable results. The method has demonstrated higher effectiveness, interpretability, efficiency and robustness compared to several state-of-the-art multivariate time series clustering methods [BDBGT22].

The modellers used the PRO symptom data, which they considered time series data (B-M12 time points) with 28 dimensions (symptoms), to cluster patients based on temporal symptom severity. In this project, we used the unsupervised mode of the Time2Feat method, which is fully automatic and uses principal component analysis (PCA) to find the symptoms that best stratify the patient cohort by symptom severity. The modellers experimented with several clusters as input for this method, from two to seven clusters. In the end, they decided to further evaluate the results for three patient clusters, which represent patient groups with mild, medium and severe symptoms. The three-cluster results showed a balanced stratification, with a 27/48/25% split. We evaluate the Bi-LSTM modelling for these patient clusters to better understand prediction patterns across cohorts.

### 4.3. Front-end design

Our front-end design comprises several user panels with coordinated components, presented below, that support the tasks involved in the two main activities (A1 and A2). Tooltips provide further details upon hovering over any component and user-selected patients are highlighted in magenta across the front-end.

#### 4.3.1 Cohort attribute distribution

**Figure 1:**
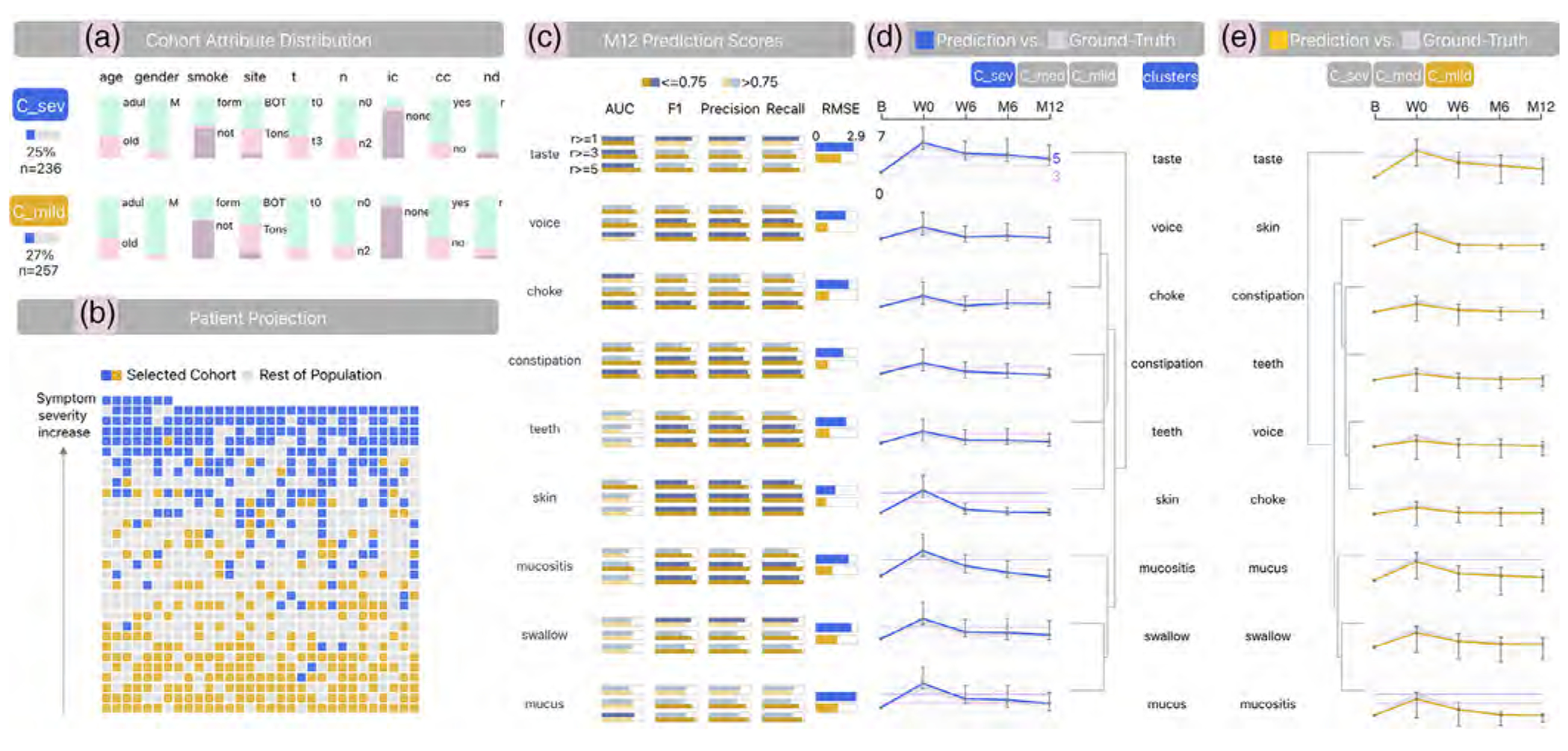
LSTM model performance on patient clusters (modeller workflow). (a) Clinical component showing two patient clusters, stratified by symptom severity. (b) Patient matrix projection (cluster with severe symptoms in blue and with mild symptoms in yellow). (c) LSTM metrics, which are similar for the two clusters; taste has the highest AUC scores for all rating thresholds. LSTM-predicted symptom trajectories for (d) the severe symptom burden cluster (blue) and (e) for the mild symptom burden cluster (yellow) against the ground truth (grey area). The LSTM model shows mild underpredictions for the first cluster and overpredictions for the second. The dendrograms show similar trajectory groupings between the two cohorts.

The cohort attribute distribution component (Figure 1a) contextualizes the cohort modelling results by allowing users to define and compare specific patient cohorts, which is an essential first step in all tasks (T1—T6). This component displays the distribution of clinical attributes, allowing for the selection of cohorts of interest for model evaluation (T1–T6). Stacked bar charts display demographic and diagnostic attributes for each patient cohort, with labels highlighting attribute values present in over 20% of the patient population. Smaller distribution values are visible upon hovering over a stacked bar. This component provides a clinical snapshot of each patient group. For the patient clusters evaluated in A1 (Figure 1a), buttons enable the selection of a cluster. For the custom, user-defined cohorts evaluated in A2 (Figure 6a), this component helps to compare attributes between a cohort of interest and the rest of the population, and dropdowns accompany the attributes to support cohort queries. We chose this compact, horizontal layout because it handles a large number of clinical attributes and easily compares different cohorts at a glance.

In Figure 1(a), the cohort attribute distribution component is used to display the clinical characteristics of the blue and yellow patient clusters selected in Figures 1(d) and (e). In Figure 6(a), the component enables the user to select the male cohort for prediction analysis, showing clinical details for both the selected cohort (top row), as well as the rest of the patient population (second row). In conclusion, the component links patient clinical details with symptom predicted and ground-truth data, shown in all the other components in both cases/figures, thus providing clinical context to all tasks in activities A1 and A2.

#### 4.3.2. Patient projection

The patient projection component (Figure 6b) is essential for grounding all modelling analysis in the actual patient population, enabling users to relate outcomes directly back to individuals or cohorts of patients during each task (T1–T6). This component uses an interactive matrix where each cell is represented by an individual patient. It represents the patient population (*e.g.* the whole dataset of patients) and it shows how cohorts are clustered. The matrix is interactive, supporting brushing a single/group of patient(s) of interest. This component is used in both activities (A1, A2) by both modellers and clinicians to relate modelling results to the actual patients during each task (T1–T6).

Blue cells highlight the patients within the selected cohort, while grey cells represent the rest of the patient population. Patients chosen directly from the matrix are highlighted in magenta. For cohort comparison (Figure 1b) (T3), the second selected cohort is highlighted in yellow. Patients are sorted by overall temporal symptom severity in a list (T5). We then populate the matrix with the patient list from the bottom left corner. In this way, low-symptom-severity patients correspond to the bottom of the matrix, and high-symptom-severity patients correspond to the top *Y* positions. The left-to-right direction (*X* axis) corresponds to an increase in severity per row. We used this projection method as opposed to others (*e.g.* PCA, t-SNE, UMAP) as it showed the best visual stratification of the patient population, with fewer outliers in the low and high symptom severity clusters extracted in Section 4.2.

In our previous work with similar patient cohorts, we have experimented with scatterplot projections to visualize the patients, either by projecting the whole population with overlapping glyphs [FNB*21], or using groups of patient projections with no overlaps on larger cohorts [FWM*23]. Rather than using a traditional scatterplot, we represent the patient population with an interactive matrix. We drew inspiration from previous work that employs matrix representations for cohorts; however, these works use matrices for cohort summaries, not for representing individuals within a cohort [ZWW*22, ZLD*15, YCB*22, KAS*20]. Our approach avoids the visual clutter and glyph overlap that often make scatterplots unreadable on large cohorts (*n* > 900), and it aims to reduce cognitive load. Because patients are consistently ordered from top to bottom by decreasing symptom severity, users can easily switch between analysing pre-defined clusters and their own custom cohorts. It also provides a scalable overview of the patient population and it could be used on larger cohorts in exchange for reducing the size of glyphs/cells.

In Figure 1(b), the patient projection method highlights the selected patient clusters in blue and yellow. This representation assists in the comparison of patient clusters (T3) showing the size of each cluster and how contrasting the clusters are from a symptom burden perspective. In Figure 6(b), the component grounds the prediction results from Figures 1(d) and (e). The component shows all the queried individuals whose predictions are displayed (males with medium taste problems at M12) to satisfy T4 and T5. It further shows that the selected cohort presents an overall high symptom burden, besides the taste problems, thus satisfying T6 as well, by uncovering further symptom severity connections to the predictions.

#### 4.3.3. Performance metrics

**Figure 3:**
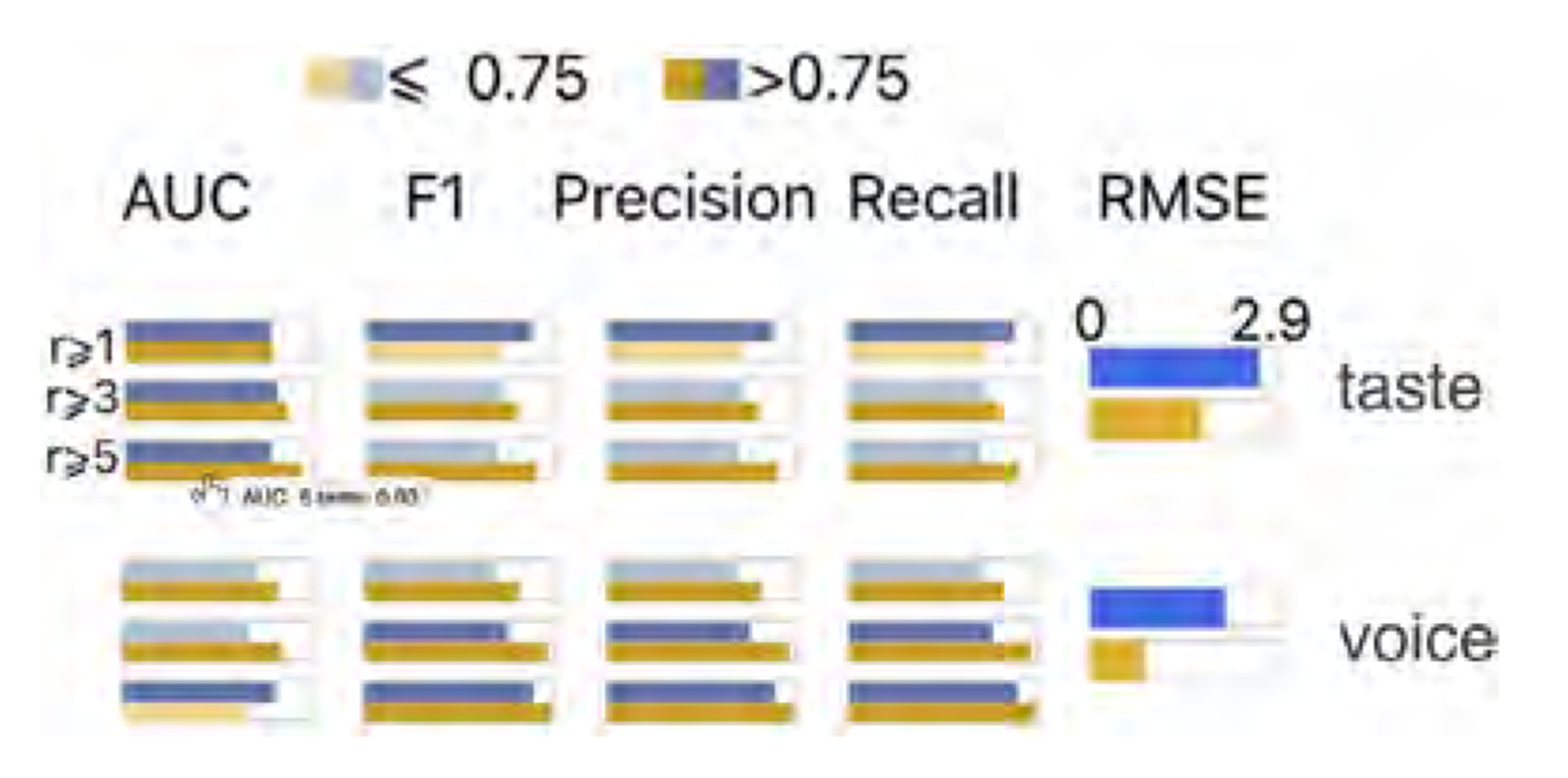
Bi-LSTM performance metrics for two symptoms at M12 time point.

The performance metrics component qualitatively validates the Bi-LSTM predictions’ accuracy under different input conditions (*e.g.* symptom severity thresholds) and identifies symptoms and cohorts where the model performs well or poorly (T3, T5, T6). We use this component (Figures 1c and 3) to evaluate the performance of the Bi-LSTM at M12 (T1). Bar plots display relevant metrics for each symptom, illustrating model performance under different rating thresholds: *r* ≥1, ≥3 and ≥5, which are clinically considered as mild, moderate and severe symptoms, respectively (Figure 3). The bar plots are rotated by 90° due to limited vertical space per symptom. We highlight good (>0.75) performance metrics (*e.g.* AUC, F1 score, Precision, Recall) with dark blue, while the rest with light blue. The RMSE metric values are not reported for rating thresholds; therefore, they are represented using the cohort’s standard colour. The 0.75 threshold is used to provide a quick visual interpretation for high and low model performance. The actual metric values are available via tooltips when hovering over each bar. The symptoms’ order is given by the first symptom dendrogram/list of trajectories (Figure 1d). For cohort comparison, we depict the values of a second selected cohort using light and dark (score > 0.75) yellow highlights. The grid-based display supports pattern and outlier detection in the metrics through the side-by-side positioning. While this component uses standard statistical charts, it displays these metrics side-by-side for different input thresholds, and the grid layout makes it easy to spot which symptoms and for what input conditions the model predicts well, and where it does not (T1).

In Figure 1(c), the component directly supports T3, by comparing AUC, F1, Precision, Recall and RMSE scores for two patient clusters alongside their predictions from Figures 1(d) and (e). For example, the performance metrics show that the Bi-LSTM can reliably separate the high- and low-risk patients for taste in both patient clusters, no matter the rating threshold, but not so well for the other symptoms, as shown by the longer and darker AUC bars. The F1, Precision and Recall scores show higher scores for the patient cluster with mild symptoms, shown by the long, dark yellow bars, suggesting that the Bi-LSTM captures lower rating predictions better and does not handle severe ratings as well.

#### 4.3.4. Symptom trajectory

**Figure 4:**
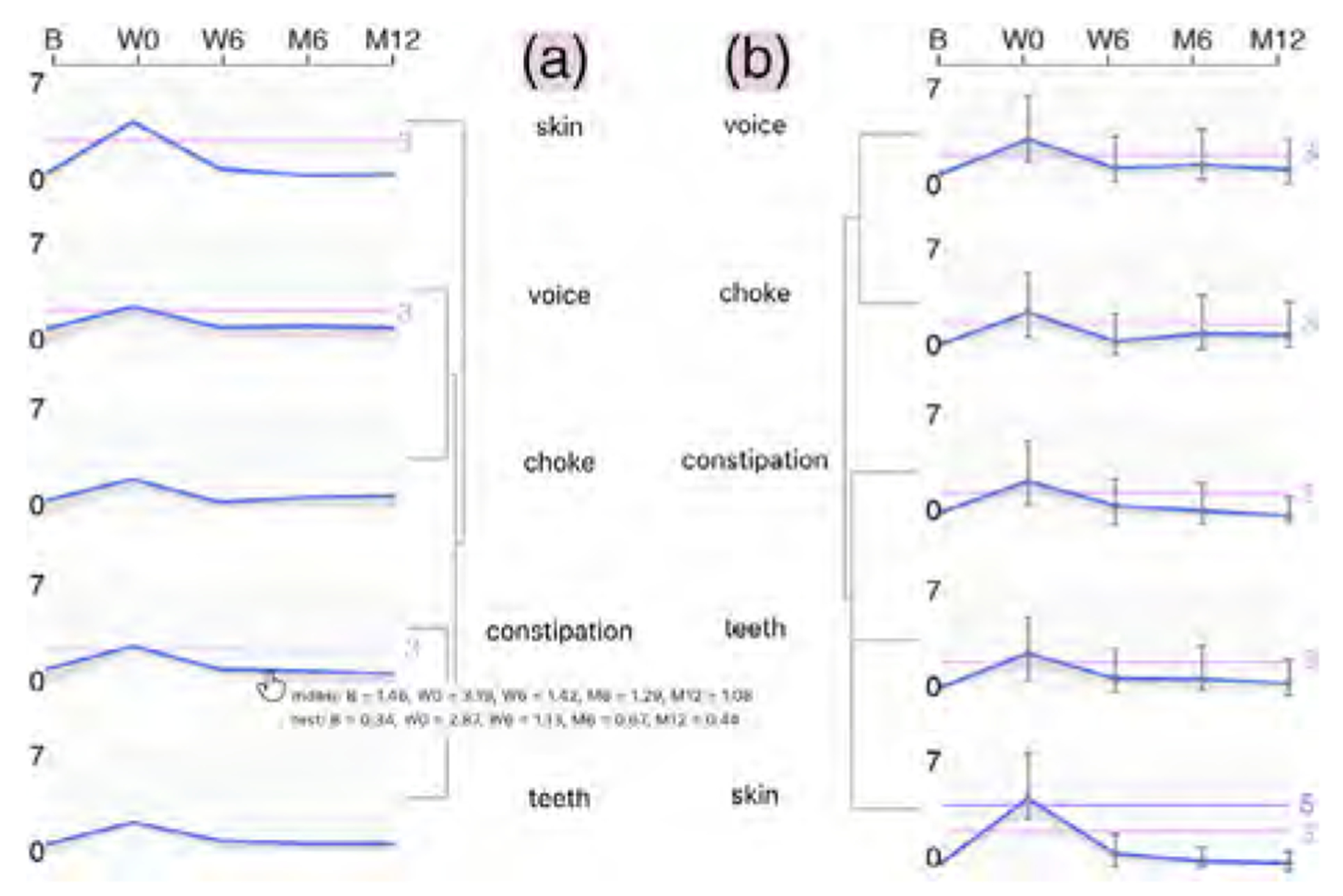
Ground-truth versus predicted symptom trajectories for the severe symptom burden patient cohort. (a) Ground-truth symptom trajectories for the cohort (blue) against the population (grey) and (b) predicted trajectories for the cohort (blue) against the population (grey) show similar symptom clusters in the dendrograms, with overpredictions at the end of treatment (W0) for all symptoms. Trajectory values span the [0,7] interval, and trajectory surpassing rating thresholds of interest (i.e. rating ≥3 for mild-to-medium severity, ≥5 for medium-to-severe symptoms) are highlighted with pink and purple threshold lines.

The symptom trajectory component (Figures 6a and b) is essential to qualitatively assess the model accuracy by comparing Bi-LSTM predicted symptom trajectories against the ground truth (T1, T3) and to evaluate the model’s robustness by examining its predictions on specific cohorts against the rest of the patient population (T4–T6) (Figures 4d and e). This component uses a lineplot to visualize how symptom severity changes over time. It can compare the model’s predictions to the ground-truth data (T1) or contrast the symptom trajectories of a selected cohort with those of the rest of the patient population (T5). The predicted values for a given cohort are represented by a blue line. At the same time, the grey area highlights the difference to the ground-truth values (T1) (Figures 1d and e) or to the predictions of the rest of the patient population (T4) (Figures 4d and e). The distribution of Bi-LSTM mispredictions is represented using vertical grey bars (upward direction for the count of overpredictions, and vice versa for underpredictions) at each time point. Brushed patients from the patient matrix projection are highlighted via magenta lineplots (T5) (Figures 4d and e). For cohort comparison (T3), the trajectories for a second cohort are depicted in yellow (Figures 1d and e). We chose this common encoding for its utility in pattern detection, as seen in other LSTM visualization work [SGPR17, HSYZ24]. Its key advantage is its versatility, which adapts to various analytical tasks for cohort time-series.

We clustered the symptom trajectories using hierarchical clustering (HC) to find consistent symptom grouping between predicted and ground-truth trajectories (T1, T4) across cohorts (T3) (Figure 4). We used Euclidean distance with the Average metric for the symptom clustering. Still, we have previously experimented with other similarity search methods for time series, such as DTW, symbolic aggregate approximation (SAX), cosine similarity and Pearson correlation, as well as with other clustering linkage methods, including Complete and Ward. These methods showed either outlier sensitivity, did not have similar trajectories within-cluster, or showed large variability in cluster formations across the patient clusters. Ultimately, we selected this method because it performed best for time series with comparable shapes and magnitudes, such as our symptom trajectories. It also did the best job of creating distinct and meaningful symptom clusters, but similar clusters across patient cohorts (T3, T5). We ordered the symptoms based on the HC results for each cohort. We used accompanying dendrograms, displayed through a mirroring technique, to represent the patterns between symptom clusters (T1, T4) for two patient cohorts (T3) (Figures 1d and e). The dendrogram dictates the vertical order of the symptoms in the other components. In the case of cohort comparison, the first dendrogram dictates the vertical symptom order in the other components (Figures 1d and e). In this way, the user can analyse a single symptom horizontally, across visual components. To reduce the cognitive load of identifying clusters and comparing trajectories across different rows in two cohorts, we offer an option to hide the symptom clusters and dendrograms. In this case, we list the symptoms in the same order across cohorts, which is based on all symptom clusters across cohorts. However, the dendrogram’s goal is to help identify highly similar symptom behaviours. This is important, given that patients generally exhibit the same overall trend in symptoms, but with different severity thresholds, and a rise in severity at W0, as a consequence of the treatment’s influence [CMW*00].

**Figure 5:**
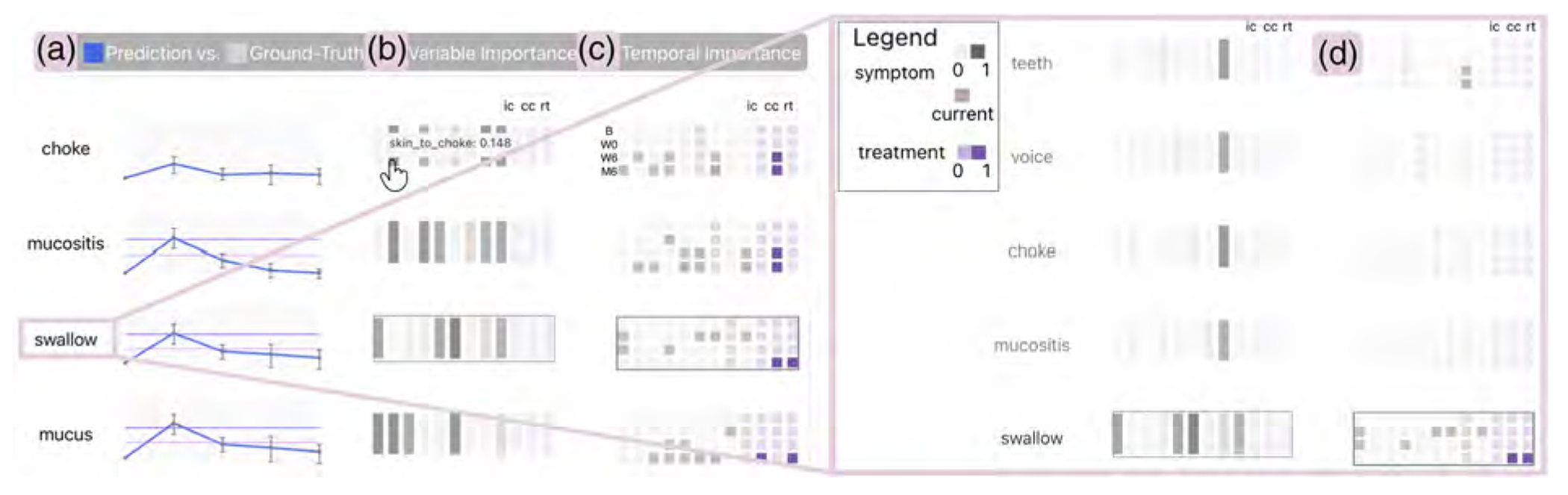
Model behaviour for the medium symptom burden patient cohort. (a) Predicted symptom trajectories with minimal mispredictions. (b) Variable importance and (c) temporal importance. Hovering on swallow’s row (d), swallow’s associations with mucositis, choke, voice and teeth are shown from (b), and high values in W6 and M6 time points (e.g. teeth) from (c), meaning associations with M12 rating prediction.

Since we visualize mainly mean values for a given cohort, we did not specify the exact numerical differences between the ground truth and the predicted values. We opted to juxtapose these differences (*e.g.* blue vs. grey or yellow vs. grey in Figures 1d and e) or to visualize trajectories side by side to compare ground truth and predicted symptoms (Figure 5). For numerical values, we provide tooltips with ground-truth and predicted values across time points upon hovering over a symptom trajectory.

**Figure 6:**
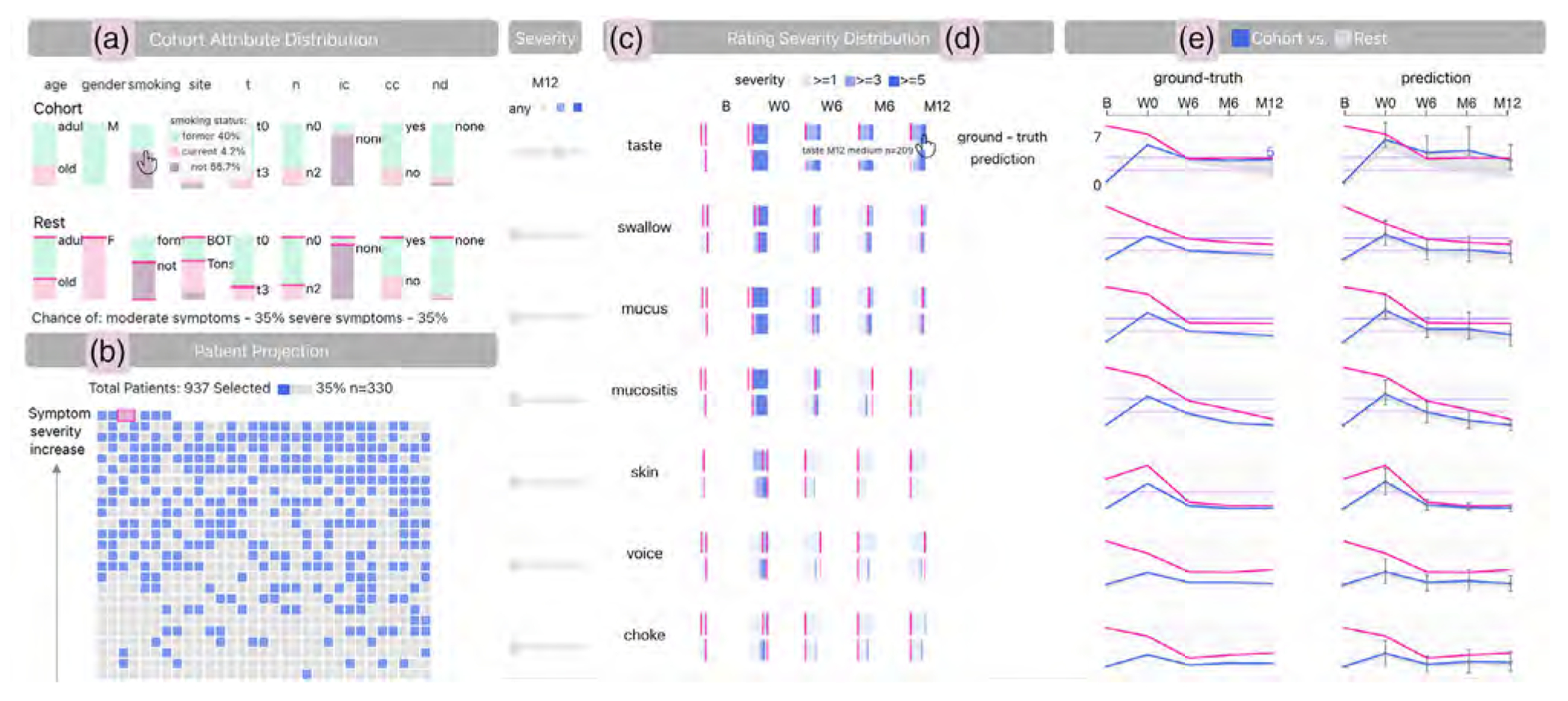
Model performance analysis for a custom cohort (clinician and modeller activity). (a) Clinical component showing the queried patients: males with medium taste problems (right c) filters) that represent 35% of the patient population. (b) Patient matrix projection based on symptom burden, showing the queried patients on the upper, more severe half, which is represented by high symptom burden. Two female outliers (grey cells) are highlighted in the projection (magenta highlight in all components). (c) Symptom severity distribution showing higher and similar prevalence for taste, swallow, mucus and mucositis. Filters for symptom severity are displayed on the left of the component. (d) Symptom trajectory for ground truth and (e) for predictions, showing differences in ground truth versus predicted trajectories at W6-M12 for taste and significant differences from the brushed patients (magenta) in all symptoms at B-W0.

In Figure 4 the component validates the predicted symptom trajectories (Figure 4b), supporting T1. The component shows Bi-LSTM sensitivity to the peak of the trajectories, as shown by the tall vertical bars from the W0 time point. The predictions show a second peak in M6 in skin and voice, which is different to the trajectory shape of the ground-truth symptoms (Figure 4a). In Figure 1, the comparison between the severe (Figure 1d) and mild (Figure 1e) patient clusters’ predictions supports T3. This comparison shows again the model’s sensitivity to peaks in trajectories. This is highlighted by the grey area in the W0 time point, which shows the difference between the prediction and the ground-truth ratings. In Figure 6, the symptom trajectories support all the tasks from A2 (T4–T6). In this example, the male patients with medium taste problems show small differences between the predicted (Figure 6e) and ground-truth (Figure 6d) trajectories, but significant differences as opposed to the rest of the patient population predictions for taste, as highlighted by the grey area in Figure 6e.

#### 4.3.5. Temporal and variable symptom importance

The temporal and variable symptom importance components are used to understand the Bi-LSTM model’s underlying mechanisms, using a complementary model (IMV-LSTM), by exposing key symptoms, treatments and time points that most influence the final time point (M12) predictions (T2).

To look inside the model‘s black box, the variable importance components (Figure 5b) explain the Bi-LSTM’s behaviour. Using importance scores from the IMV-LSTM, it highlights which features (*i.e.* symptoms and treatments) contribute most to the prediction of a given symptom (T2). We can see the variable importance as weighted associations between the symptoms and treatment type and the M12 prediction for a given symptom. We use a matrix representation to show the variable importance of each symptom to predict a given symptom. Each row lists the symptoms based on the symptom list from the symptom trajectory component, and it visualizes the mean variable importance of a cohort for each symptom. The same order is followed in the columns. Brown corresponds to the current symptom, a grey colour scheme is for the rest of the symptoms, while a purple colour scheme is for the global importance of the treatment types. Lighter colours and lower opacity correspond to lower variable importance, while darker colours and higher opacity encode higher values. For each symptom, we highlight reliable results, characterized by a high AUC (>0.75), using a dark margin around the corresponding symptom’s row. When hovering over a symptom label, the corresponding variable importance is highlighted in the row (Figure 5d) to illustrate how the symptom affects the predictions of other symptoms. During hovering, the column corresponding to the given symptom is highlighted to show the influence of the other symptoms in the prediction of the given symptom. The design is inspired by previous work for LSTM hidden states visualization [SGPR17]; however, we visualize both variable importance and temporal importance for each symptom. Moreover, we took inspiration from related work on cohort summaries using matrix encodings [ZWW*22, YCB*22, KAS*20, ZLD*15], but we highlight which items (*i.e.* symptoms) show reliable associations with other items through the rows’ dark margins.

The design of the temporal importance component mirrors that of the variable importance component (Figure 5c). This consistency makes the temporal importance analysis more intuitive and easier to follow (T2). It shows the IMV-LSTM-generated importance of the symptoms’ time points (*i.e.* B-M6) in predicting the M12 rating for a given symptom. In other words, we can see the temporal importance as a weighted association of the symptoms’ time points to the prediction of the M12 rating. Following the matrix-based design, the row visualizes the mean temporal importance of a cohort for a symptom. Each row is split into smaller cells by time points on the vertical axis. The same interactions to the variable importance component apply here as well. Upon hovering over a symptom, the row highlights the influence of the given symptom to the rest. The highlighted corresponding column shows the influence of the other symptoms on the hovered one.

In Figure 5, the variable (Figure 5b) and temporal (Figure 5c) importance support T2 by exposing the Bi-LSTM memory. In this example, we see that the prediction for swallow relies on the ratings from associated symptoms, which are highlighted upon swallow’s hovering, namely, teeth, voice, choke and mucositis. The last two symptoms show associations in W0 with swallow’s prediction as well (Figure 5d 6th and 7th columns on the right side.

#### 4.3.6. Symptom severity distribution

The symptom severity distribution component compares predicted symptom distributions against the ground-truth over time, which helps to test the model’s accuracy (T1, T4) and find cohorts and symptoms for which the model predicts high-risk outcomes (T5, T6). In a similar fashion to the performance metrics component (Figure 1c), this component (Figure 6c) uses a grid-based representation to display the temporal severity distributions. The top rows represent the ground-truth severity distributions and the bottom rows the predicted symptom distributions (T1, T4). With a similar design to the cohort attribute distribution component (Figure 4a) each cell is represented by a stacked bar chart showing the distribution of symptoms (rating > 0), with light-to-dark blue colours representing mild (*r* ≥ 1), medium (*r* ≥ 3) and severe (*r* ≥ 5) ratings. The horizontal bars support the side-by-side comparison between the ground-truth and predicted values. Numerical values are provided by tooltips upon hovering on the distribution rows. For custom cohort model analysis (A2), each symptom is accompanied by a severity slider which filters the patients with the corresponding severity for the last time point, M12 (T5). The component highlights patterns in symptom presence and shows which symptoms are severe and need more attention during analytical workflows (T6). Brushed patients from the patient matrix projection are highlighted with magenta borders in this component. We chose this grid layout over other chart types because it provides a compact and efficient display of temporal, multivariate data. Other designs were considered, but would have occupied more screen space, such as box/violin plots and pie charts. This grid layout facilitates item (symptom) comparison, as well as comparison of temporal and data provenance (ground truth vs. predictions).

In Figure 6c, the symptom severity distribution component compares the predicted and ground-truth symptom severity distributions for the selected cohort (males with medium taste problems in M12), thus supporting T4. As a consequence of the filters used to query this cohort, taste shows the highest overall severity across time and data provenance (*i.e.* ground-truth and predictions). The component shows further connections between the taste severity threshold and the rest of the symptoms’ predictions, some of which have high and medium severity in W0 (T6).

### 4.4. Workflows

The stratified model evaluation activity (A1), supports modeller tasks using predefined workflows represented by several panels. These panels are used to validate the Bi-LSTM and IMV-LSTM results on three patient clusters. An example is presented in Figure 1, where the Bi-LSTM results are validated (T1) and compared (T3) between the patient clusters with mild and severe symptom burdens. The selection of the two clusters from the clinical component (Figure 1a) is linked to the other components. Specifically, the clusters are highlighted in the projection in blue and yellow (Figure 1b), their corresponding prediction metrics are represented in the corresponding component (Figure 1c) (T1) and their predictions are compared to the ground truth on symptom trajectories (Figures 1d and e) (T3). Alternatively, another workflow examines the Bi-LSTM memory exposition to understand the model’s behaviour (T2) and its prediction decisions by analysing the IMV-LSTM temporal and variable importances (Figures 5d and e) for a given patient cluster (Figure 5a).

A third panel supports the targeted model evaluation (A2), on user-defined cohorts (Figure 6). This configuration enables both data modellers and clinicians to analyse modelling results for a specific cohort of interest. This workflow provides fewer model behaviour details (*e.g.* no memory exposition) to lower the cognitive load during clinician analyses. The user can better understand how sensitive predictions are on diverse patient attribute inputs (T4) once they select a cohort from the cohort attribute distribution component (Figure 6a). The selection is linked to the other components: The cohort is highlighted in the patient matrix projection (Figure 6b) in blue, its symptom severity distribution is represented on the right (Figure 6c) (T5), which connects symptom presence with prediction results. The selected cohort’s ground-truth (Figure 6d) and predicted (Figure 6e) trajectories, compared to the rest of the patient population (T5), are further represented on the right side of the panel. Further brushing/selection on the patient matrix projection will highlight the corresponding patients in magenta. This panel and activity provide a common ground for hypothesis generation in clinician–modeller collaborations.

## 5. Evaluation

In this section, we describe the evaluation methodology, which includes two case studies, and the feedback from our evaluators.

We evaluated L-VISP qualitatively through demonstrations and case studies with two data modellers, the visual computing team and a senior research oncology expert, who had ML experience. The data modellers participated in the design of the visual analytics system and in the model building, and the oncologist provided occasional input and feedback. All evaluators are co-authors. Although the system serves modellers in cancer research, validating the results with a clinician was essential to ensure their clinical relevance.

The evaluation was based on pair analytics [AHKGF11], where the main visual analytics designer was the navigator of the visual analytics tool (L-VISP) and the collaborators were the drivers of the tool. Although pair analytics requires two participants per session, we organized group evaluation sessions due to the collaborators’ limited availability. Additionally, we observed that group sessions helped to generate more hypotheses and feedback. However, these sessions usually had two main drivers, namely a data modeller and the clinician. The evaluation was conducted online, through screen sharing, starting with demonstrations of the tool and then walking through case studies. The drivers (evaluators) were encouraged to think aloud and make hypotheses while the navigator was driving the interface, and a navigator helper (from the visual computing team) was taking notes. The data modellers presented two case studies, described below, on a cohort of 937 HNC patients treated at the MD Anderson Cancer Center, and the clinician validated the results.

### 5.1. Blended models insights and evaluation

In the first case study (A1), the modellers were interested in evaluating the symptom modelling results on the pre-computed patient clusters (Figure 1) and getting insights into how the Bi-LSTM makes predictions (A1). The patient matrix projection revealed that the clusters were separated by temporal symptom severity into the severe (top), the medium (centre) and the mild cluster (bottom) (Figure 1b). Selecting the severe patient cluster, the modellers observed that it consistently showed higher severity in predictions as opposed to ground-truth data across all time points and all symptoms (Figure 4) (T1). The modellers observed that most overpredictions occurred in W0, which was typically the highest-rated time point, and noted this biased result for future refinements. The symptom clusters highlighted by the dendrogram were similar between the ground-truth and predicted symptoms (T1, T3) (Figure 4), showing that the model captured the same temporal patterns as the ground truth (T1). The Bi-LSTM predictions revealed three consistent symptom clusters across patient clusters, with ‘taste’ as the first cluster; Figure 4 representing the second and ‘mucositis, swallow and mucus’ as the third (T3).

There were more high AUC and F1 score values for the mild patient cluster, suggesting that the model captures lower symptom ratings more effectively (Figure 1c) (T3). The highest RMSE was observed for M12 in the taste prediction, while the lowest was for skin problems. This was verified in the timelines (Figures 1d and e) where skin consistently had the lowest mean in M12, suggesting that a lot of patients might not report skin (T1), while taste had the highest mean, which was expected as taste has shown to be one of the most prevalent symptoms in our previous symptom research work [FWM*23]. The modellers and the oncologist agreed that taste showed more severe and persistent patterns *‘We see that taste is its own thing’*; *‘I am not surprised taste is so common’*. The dendrograms highlighted similar symptom clusters for both the mild and severe patient groups (T3). This finding, despite the groups’ differing severities, suggested that symptoms have consistent trajectory groupings regardless of severity. When checking the Bi-LSTM performance across clusters (Figure 1), the model consistently showed overprediction for the severe cluster, and underprediction for the mild cluster, across all symptoms (T1, T3) (Figures 1d and e). The modellers agreed that the model tends to be more sensitive to severity extremes in the patient population.

The hidden states of the Bi-LSTM black box (Figure 5) showed that only a couple of symptoms were highlighted as reliable predictions (T2). Swallow unsurprisingly showed associations with predictions for symptoms connected to the salivary domain (Figure 5d). However, its association with teeth was an unexpected finding, which the oncology expert suggested needed to be further investigated *‘it’s hard to tell the root cause of tooth pain, it can be from choking or pain, or a reflection of mucositis problems’*. The temporal importance (Figure 5c) showed that most of the symptoms tend to be associated with M12 predictions at the end of the patient observation period, in W6 and M6 (T2), but did not show any common symptom patterns with the variable importance, which was surprising to the modellers.

### 5.2. Model output analysis for targeted cohorts

In the second case study (A2), the modellers, together with the clinician, explored the cohort to evaluate how the model predicted symptoms in subsets of patients (Figure 6) (A2). They retrieved the male patients (Figure 6a) with medium taste problems in M12 (Figure 6c). This selected cohort is displayed at the top of the patient matrix projection (Figure 6b), where patients with higher overall symptom severity were displayed. When selecting two outlier females against this cohort (Figure 6b) (T4), the clinician observed some high Bi-LSTM predictions at M12 for swallow, voice and choke, suggesting that the brushed patients show a higher risk for these symptoms (Figure 6e) (T5, T6). The modellers recorded the patients’ IDs of these outliers for further investigation.

Next, the evaluators checked how the Bi-LSTM model performed on the selected cohort (Figure 6c) (T5). The system revealed that the selected cohort consistently showed higher mean ratings for both the ground-truth and predicted values compared to the rest of the patient population (T4, T6), as well as higher trajectory ratings (Figures 6d and e). An interesting pattern was observed in the Bi-LSTM trajectories across symptoms that showed increases in M12, as opposed to the rest of the population, such as taste and mucus (T6). The Bi-LSTM outputs showed a second peak in M6 for these symptoms, suggesting that the model, by learning from both temporal directions, detected the increases in M12 in the ground-truth data (T5). This was not obvious in the preliminary analyses of the model results.

The clinician expressed that looking at symptom statistics for the desired cohort is what he is mostly interested in *‘Summaries of chances of having anything (symptoms) over five (rating)’*. He also added that this activity would benefit his clinician colleagues to analyse cohorts of interest.

### 5.3. Expert feedback

The evaluators’ feedback was extracted from meeting notes and direct written feedback. We performed a reflexive thematic analysis [BC21] on the feedback and extracted common themes.

#### Perceived usefulness.

The modellers’ feedback showed that L-VISP is valuable in their research practices: *‘There is so much output data generated […](L-VISP) is instrumental in facilitating the exploration of those outputs, comparing the performance of different patient groups, and visualizing the temporal symptoms importance. In the targeted evaluation, we can use patient filters that allow for hypothesis testing. The IMV-LSTM generates summary figures […] these vary greatly between different cohorts, and it would not be possible to identify these differences without this’* and *‘I appreciate how intuitive the system can show and compare Bi-LSTM’s performance among different symptoms and patient cohorts’*.

#### Actionability.

The feedback showed that L-VISP is fit to be used for clinical research. One modeller expressed:*‘It helps me a lot to analyze and understand the behavior of the Bi-LSTM. The compact, yet informative, representation of […] allows us to see not only which variables contribute to the target symptom, but also how important one symptom contributes to all other symptoms’*. The clinician appreciated the possibility to analyse cohorts of interest *‘I am interested in seeing simplified probabilities of severity, such as toxicity at X months… (given a cohort) which this supports’*.

#### Trust.

Statements from our evaluators regarding L-VISP’s actual use in practice and agreeing on hypotheses during the evaluation showed that they trust the system’s results. The oncologist expressed that they were considering showing the L-VISP results to their patients and coworkers: *‘I can show these to my colleagues and even my patients’*. Furthermore, the modellers agreed during one of the case studies that *‘The LSTM overpredicts for the severe patient cluster and underpredicts for the mild cluster’*.

## 6. Discussion

L-VISP highlights symptom patterns and groupings generated by LSTM modelling that extends previous research in head and neck cancer post-treatment [FWM*23, FNB*21, WVDM*23, WCVD*21]. Our evaluation showed that L-VISP can blend results from multiple models, enabling tasks that range from evaluating Bi-LSTM performance on patient clusters (Figure 1) to visualizing hidden states from the IMV-LSTM (Figure 5) for deeper model insights. L-VISP helped the modellers capture input–output relationships in the Bi-LSTM results, showing increasing trends in targeted patient cohorts with severe symptoms (Figure 5). Our visual system compared performances between cohorts and revealed that the Bi-LSTM showed consistent predicted symptom clusters among cohorts (Figures 1d and e). L-VISP validated the Bi-LSTMs predictions, revealing mild mispredictions for the patient clusters with severe and mild symptoms (Figure 4).L-VISP was able to capture insights into the Bi-LSTM decision-making by revealing associations between symptoms during prediction (Figure 5). The modellers expected to see more reliable patterns in the model’s behaviour, which was not the case, but were overall content with the post-treatment symptom predictions.

L-VISP was developed mainly for data modellers to create interpretable models in clinical practice. Through expert feedback and generated hypotheses, our evaluation shows that modellers can effectively summarize cohort modelling results and collaborate with clinical experts to clinically interpret the models. While our case studies target head and neck cancer patients, we generalize our design to multivariate, temporal patient cohorts where the focus is to evaluate and compare different model outcomes against ground-truth data for various cohorts. We generalize most of our design choices to other fields that need complex temporal prediction output interpretation in multidisciplinary collaborations with multiple workflows. Specifically, L-VISP can support other variants of the LSTM family for temporal predictions. The ACD collaborative and iterative approach ensured that L-VISP met technical requirements for data analysis and aligned with our collaborators’ workflows. The ACD design process revealed a key insight for clinician–data modeller collaborations, which was to visually separate the results presented to modellers and clinicians, although the clinician would analyse the results together with the modeller. Specifically, we separated model debugging tasks from clinical model interpretation tasks. Below, we present a couple of lessons learned from this multidisciplinary collaboration.

### L1. Visually separate activities.

During the software prototyping phase, we experimented with different front-end layouts and separated the interface into three configurable panels. However, our collaborators had difficulties configuring the panels to support a particular analytical flow. Instead, we revised the design to provide predefined layouts, each designed based on a main activity, and a navigation bar to switch between activities.

### L2. Reduce visual information density when necessary.

L-VISP was originally designed on a large display, and incorporated modelling results for all 28 symptoms. However, this resulted in a high data density, and the visual encodings and text became illegible. As a result, we redirected our efforts toward interpreting the modelling results for the symptoms of primary interest, namely the nine HNC symptoms. With the reduced information, we increased the scale of the visual encodings and the text labels. We also streamlined our labels. Following this design update, the modellers reported that they could see the information more clearly and form hypotheses more quickly.

### L3. Re-use visual components for multiple activities.

Given the project requirements and multiple workflows, we minimized variability in the visual component design, and reused components for different purposes. This design choice was made to lower cognitive load for end-users with different modelling expertise (*i.e.* modellers vs. clinicians) and to keep consistency across activities. This resulted in a lower learning curve for data modellers, enabling the clinician to quickly interpret results.

Design limitations include the inability to legibly visualize data for all 28 symptoms, such as the symptom trajectories, the variable importance and temporal importance components (*i.e.* would require vertical scrolling). L-VISP does not support more than two-cohort comparisons, which in turn supports legible LSTM outcome visualization. The clinical component can support a limited number of attributes and sub-cohorts/clusters (*i.e.* would require horizontal scrolling) in the cohort attribute distribution component. On the other hand, the patient matrix can support 2D projections based on other combinations of attributes, and a larger cohort (*i.e.* thousands) at the cost of limiting individual patient selection/brushing. On large cohorts with tens of thousands of patients, the system can visualize the summarized LSTM results and the three-cluster stratification. However, individual patients depicted in the projection matrix would not be legible.

Future work will address updating the current design to scale for all 28 symptoms and for larger patient cohorts. Visual clustering could be an alternative to individual patient and symptom visualization. Another direction would be to update the Bi-LSTM model to address the issues identified during our evaluation (*e.g.* the overpredictions for the severe patient cluster and underpredictions for the mild cluster). This could help the data modellers to use the model on future patient cohorts. Natural language queries would be a faster option for generating the desired workflows for clinicians or data modellers. The queries would configure the front-end with appropriate visual components (*e.g.* ‘Show LSTM predictions’) for the desired cohorts (*e.g.* ‘for female patients that had neck surgery’). This last extension would enable faster model debugging. As the clinician showed interest in using L-VISP with his colleagues, this direction could better incorporate clinician workflows that focus on prognosis for new patients.

## 7. Conclusion

In conclusion, we described L-VISP, a human–machine solution that supports cohort modelling by enhancing the interpretation of symptom risk models for head and neck cancer patients. Our domain characterization for black-box cohort risk modelling revealed the different user workflows in interdisciplinary clinician–data modeller collaborarons. Our proposed visual analytics system utilizes predefined layouts while integrating multiple cohort modelling methods to support various analytical workflows. We use custom visual encodings to explain model behaviour and to evaluate model performance across cohorts. The generated hypotheses and the collaborative analysis during the evaluation with data modellers and a clinician shows the usefulness of our approach for interpretable visualizations in model building for clinical applications.

## Figures and Tables

**Figure 2:**
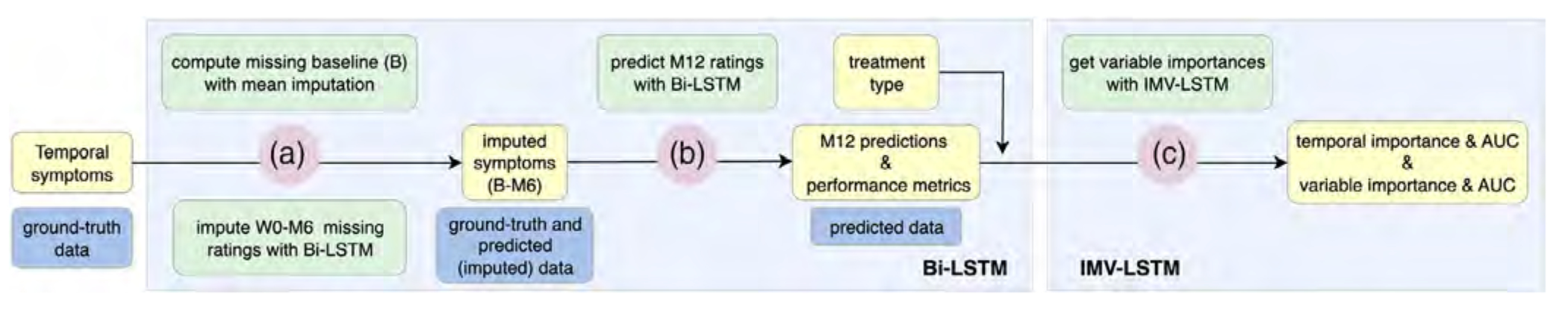
LSTM symptom modelling pipeline. (a) Imputation of missing symptom ratings, for all time points up to the last one (M12) using Bi-LSTM; except for the B time point, which is imputed using mean B imputation. (b) Bi-LSTM prediction for symptoms at the last time point, M12. (c) Application of IMV-LSTM on the Bi-LSTM imputed values to extract temporal and variable feature importance for all symptoms.

**Table 1:** Symptom ratings example for two patients (P1, P2) and for two symptoms (taste, swallow) over time (B for baseline, W0 for first week post-treatment, W6 for 6 weeks post-treatment, M6 for 6 months post-treatment and M12 for 12 months post-treatment). Ratings are reported on a 0–10 scale, from ‘not present’ (0) to ‘as bad as you can imagine’ (10).

	Taste B	Taste W0	Taste W6	Taste M6	Taste M12	Swallow B	Swallow W0	Swallow W6	Swallow M6	Swallow M12
**P1**	0	4	6	5	4	1	5	6	7	5
**P2**	2	3	4	6	5	0	0	4	3	3

## CCS Concepts

• Human-centered computing → Scientific visualization; • Computing methodologies → Machine learning; • Applied computing → Life and medical sciences;
